# 

**Published:** 1894-08-11

**Authors:** 


**Notices of Births, Deaths, and Marriages** are inserted in this column at a charge of 2s. 6d. for an announcement not exceeding four lines.

**Notice to Advertisers and Subscribers.**

All Advertisements, Orders for Copies of The Hospital, and Business Communications should be addressed to **The Publisher, NOT TO THE EDITOR,** The Hospital, 428, Strand, W.C.

The Cost of Subscription, post free, is as follows:—Per week, 2½d.; per quarter, 2s. 8d.; per half-year, 5s. 3d.; per annum, 10s. 6d. Foreign:—Per Annum, 12s. 8d.; payable, in all cases, in advance.

### NOTICE TO CORRESPONDENTS.

All MS., Letters, Books for Review, and other matters intended for the Editor should be addressed The Editor, The Lodge, Porchester Square, London, W.

The Editor cannot undertake to return rejected MS., even when accompanied by stamped directed envelope.

**"Burdett's Hospital Annual," 1894.**

Fifth year of publication. 900 pp. Boards, gilt lettering. A most complete guide to British, Colonial, Indian, and American Hospitals, Dispensaries, Nursing and Convalescent Institutions, and Asylums. Price 5s. Published by The Scientific Press (Limited), 428, Strand, W.C.

**NOTICE.—The Index for Vol. XV. (ending March 31st, 1894) is on sale at the Office of this Journal, price 2½d., post free.**

### SCRAPS AND GLEANINGS.

Hospital Saturday collection at Brighton reached this year to the amount of £1,150.

During last month 899 patients were treated at the Royal Ear Hospital, Frith Street, Soho Square.

On the recommendation of the Council of the Royal College of Physicians the Moxon medal has been awarded to Sir William Jenner.

At the request of the Inspectors of Irish Asylums, the Governors of Limerick Asylum have agreed to provide accommodation for 160 more patients.

The Great Northern Central Hospital, Holloway, which was in great need of funds, has received a donation of £100 from the Grocers' Company.

Founders' Day at Epsom Medical College was observed on Monday, when prizes were distributed and speeches made by various members of the Council.

The new casual wards which have been built by the St. Saviour's Guardians at Southwark are now completed, and will be opened as soon as the staff has been appointed.

Dr. Fleming, late principal veterinary surgeon to the army, has been appointed honorary president of section 17 of the International Congress on Hygiene to be held at Budapest in September.

The committee and friends of the Cork Eye, Ear, and Throat Hospital held a meeting last month in order to make arrangements for a great bazaar which is to be held in Cork in October in aid of the hospital.

The managing board of the Rotherham Hospital and Dispensary are soliciting the aid of the inhabitants in and near Rotherham in defraying the expenses of a new boiler and heating apparatus for the hospital.

A conference has been held of urban and rural authorities at Bury to consider the proposal to have a joint hospital for infectious diseases for Bury, Radcliffe, Ramsbottom, and Whitefield. The scheme is still under discussion.

The Women's Volunteer Medical Staff Corps is making steady progress. A course of lectures is to be commenced this month with a view to giving instruction to the corps in the technical points of army ambulance work.

At the annual meeting of the Corbett Hospital at Stourbridge it was shown that a children's ward is urgently needed, and it was suggested to adapt the room in which the meeting was held for the purpose, at an expenditure of about £200. The report was adopted.

Dr W. G. Willoughby suggested at the Health Congress that a large and separate block of the Borough Hospital should be given up entirely to the schools as an infectious diseases hospital, and that the school physicians should be allowed to attend their own cases.

The prize festival and twenty first annual meeting was held last week at the Royal Normal Hospital and Academy of Music for the Blind at Norwood. A concert was given and glees rendered by a blind choir. The earnings of old pupils of the college last year amounted to £20,000.

A meeting has been held at Omagh for considering the advisability of erecting a new infirmary for the county. It was unanimously decided to appoint a committee to collect subscriptions without delay for that purpose. The present building is over 100 years old, and shows signs of decay.

A new fever hospital is about to be erected by the Metropolitan Asylums Board at Shooter's Hill. The cost of the new hospital is estimated at £200,000. The site comprises 32 acres, and accommodation will be provided for 500 patients suffering from enteric fever or diphtheria.

A garden party and fête were lately held at Farley Lynches in aid of the Bute Hospital, and prizes were given for table decorations, bouquets, and baskets of flowers. There was a children's floral parade, and a concert was held in the evening, after which there was dancing on the lawn.

At the concluding meeting of the Public Health Congress the attention of the Board of Trade was called to the fact that there is no arrangement made for the enforced periodical cleansing of railway carriages, and it was suggested that some rules should be made to bring the matter under sanitary control.

The sanitary officers in Hong Kong meet with a poor reception at the hands of the populace, who violently oppose any sanitary measures to suppress the present epidemic of so-called plague. In several instances the officials have been stoned in the course of their door-to-door inspection.

The Royal College of Surgeons having passed a regulation requiring medical students, previous to their final examination, to produce certificates of having attended clinical demonstrations at a recognised lunatic asylum, the new asylum at Claybury has been selected as the most convenient place for pathological study of this nature.

At the annual meeting of subscribers to the Swansea General Hospital, the accounts showed an adverse balance of £1,638 17s. 10d. It is hoped, however, that the proceeds from the grand entertainment provided by Mr. Studt, and those of the concert given by Madame Patti, will go far to retrieve the financial position of the hospital.

The total amount paid into the Hospital Saturday and Sunday Fund at Southampton amounts this year to £1,142 10s., being rather less than last year. A large contribution was collected from the boats of the Union Steamship Company, and it is hoped that next year the other vessels in port will make collections on behalf of the fund.

A convalescent home for the county of Leicester was opened in July. The hospital is situated in the Charnwood Forest, and the site is admirably selected for convalescents. The committee have provided for 30 patients, but it is proposed to extend the privileges of the home to patients who can pay a fee of one guinea per week.

The Public Health Committee of Edinburgh are looking about for a favourable site for the erection of an emergency fever hospital. The Mansion House and grounds of Parson's Green they find could be converted into a hospital at a small expense. The inhabitants of the district are, however, already protesting against the scheme.

### The Student of Medicine.

### APPOINTMENTS.

Mr. G. Granville Bothwell, appointed House Surgeon at Croydon General Hospital. H. R. S. Clarke, M.R.C.S., L.R.C.P.Lond., appointed Assistant Medical Officer to the Infirmary of the Parish of Birmingham. J. H. Dewhurst, M.A., M.B., B.C.Camb., M.R.C.S., L.R.C.P.Lond., appointed Medical Officer for the Campden District of the Shipston-on-Stour Union. E. D. Duffett, L.R.C.P., L.R.C.S.Edin., appointed Medical Officer for the Illogan District of the Redruth Union. Dr. Fullarton, appointed Visiting Surgeon for the Treatment of Diseases of the Throat, Nose, and Ear at the Greenock Eye Infirmary. G. Heaton, M.A., M.B., F.R.C.S., appointed Honorary Surgeon to the General Hospital, Birmingham, in place of the late Mr. Robert Jolly. Mr. W. A. Payne, appointed Medical Officer for the No. 7 District of the Barnstaple Union. G. R. Wilson, M.B., C.M.Edin., appointed Medical Superintendent at the Mavisbank Asylum, Lasswade. S. W. Wilson, L.R.C.P.I., L.R.C.S.I., appointed House Surgeon to the Dorset County Hospital, Dorchester. G. E. Yarrow, M.D.Heidelb., L.R.C.P.Lond., M.R.C.S.Eng., appointed Deputy Coroner for the North-Eastern Division of the County of London.

### VACANCIES.

### Resident Medical Officers.

Bath Eastern Dispensary.—Resident Medical Practitioner. Salary £100 per annum, with furnished apartments, coal, gas and domestic attendance. Applications to Colonel F. V. Eyre, R.A., Honorary Secretary, Rockville, Lansdown, Bath, by August 11th.

Hants County Asylum.—Third Assistant Medical Officer. Salary £100 per annum, with furnished apartments, board, washing, and attendance. Applications and testimonials before August 23rd to Committee of Visitors, Knowle, Fareham.

Liverpool Infirmary for Children.—Assistant House Surgeon. Board and lodging, but no salary. Applications and testimonials to C. W. Carver, Hon. Secretary, by August 18th.

Sussex County Hospital, Brighton.—Assistant House Surgeon, unmarried, and under 30 years of age. Emoluments are a salary not exceeding £30 per annum, with board, washing, and residence. Applications to the Secretary by August 15th.

West Riding Asylum, Wakefield.—Fourth Assistant Medical Officer. Salary £100 per annum, rising £10 annually to a maximum of £150, with furnished apartments, board, washing, and attendance. Applications to the Medical Director by August 18th.

### Non-Resident Medical Officers.

Aberdeen Royal Infirmary.—Ophthalmic Surgeon. Applications by August 31st.

Burgh of Leith.—Medical Officer of Health. Salary to commence at £350. Applications and testimonials to T. B. Laing, Town Clerk's Office, Leith.

THE STUDENT OF MEDICINE—*continued.*

**PASS LIST.**

**Royal College of Physicians, Edinburgh, Royal College of Surgeons, Edinburgh, and Faculty of Physicians and Surgeons of Glasgow.**

The Quarterly Examinations for the Triple Qualification in Edinburgh took place in July, with the following results:

First Examination, Four Years' Course.—Of 15 candidates the following 7 passed: M. O'Brien, J. Levents, R. M. Powell, J. W. Norwood-Hudson, R. F. Noyes-Overton, J. McLachlan, and J. McElroy. Of 4 candidates who entered for the respective divisions 3 passed.

Five Years' Course.—Of 28 candidates the following 19 passed: Margaret G. C. Brodie, C.F. Ackland, Faith Philpot-Crowther, D. Graham, Harriette F. Bailey, M. H. Babington, Elsie R. C. Taylor, Mathilda H. G. Russell, J. Hope, J. K. Calder, J. P. Owens, D. Heron, Alice Hutchison, Jane M. L. Foley, W. S. Soutar, E. L. Munn, C. J. Greig, Tina McCulloch Alexander, and Jane A. Craig. Of 9 candidates who entered for the respective divisions 6 passed.

Second Examination, Four Years' Course.—Of 46 candidates, the following 27 passed: E. B. Hicks, H. Holden, J. W. Norwood-Hudson, H. J. M. Wyllys, G. C. Henry, W. D. McMurtry, E. F. de Jong, J. Minns, R. M. Johnson, H. S. O'Connor, R. M. Powell, C W Holmsted, E. Pearson, G. G. Irving, J. E. O'Ryan, T. F. Elmes, H. O'Donohoe, S. G. A. Jeffs, H. Highet, W. J. Spearing, T. Homer, W. J. Hayes, F. E. Richardson, G. A. I. Mackay, Beatrice A. McGregor, D. Hennessy, and J. D. Duhig. Of 4 candidates who entered for the respective divisions 3 passed.

Five Years' Course.—Of 21 candidates, the following 20 passed: J. St. J. Murphy, R. W. Mickle, J. M. Donovan, A. J. Laurie, E. C. Macintosh, Eliza McFarlane, E. B. Kitching, Elizabeth M. Johnson, P. Pearse, W. Robertson, J. Murray, A. Cameron, Robina McGregor, Euphemia Cumming, Janet D. Gilfillan, Helen W. Stanley, Georgina C. Hogg, Christina A. Mayne, Mary A. Stewart, and A. F. Jones.

Final Examination.—Of 115 candidates, the following 62 passed and were admitted L.R.C.P.E., L.R.C.S.E., and L.F.P. and S.G.: M. Wilson, D. D. Muir, J. Russell, E. A. R. Covey, T. H. Agnew, F. G. W. Deane, C. H. B. Armstrong, G. E. Douglas, A. Harry, P. Slack, T. Taylor, H. H. Osborn, W. Corkey, W. L. Crewdson, W. E. Bracey, J. Nelson, R. Dagger, H. Gillies, E. C. Hayes, A. O'Leary, C. S. Edwards, J. W. McGregor, G. G. Vatve, Ida Bowie, F. J. Farley, M. M. Joseph, Florence A. Holt, W. Dill, G. Gibson, J. G. D'Mello, D. F. Blanchard, H. M. M. Mackenzie, P. Sheehan, E. R. A. MacDonnell, W. H. Penrose, C. E. Player, K. V. Kukday, W. G. Donald, J. B. D. St. Cyr, A. Duncan, J. F. Campbell, T. O. Jones, C. L. Hayes, Holland M. Harrison, J. J. Hayes, H. P. W. Lincoln, E. J. H. Budge, J. J. Bell, T. D. Sullivan, F. A. Routch, F. S. Hogg, W. H. Thomson, D. H. Scott, L. Williams, W. P. McGrenahan, J. F. Sutcliffe, J. S. Gallie, J. O'Leary, W. C. Gent, F. G. Smith, P. J. O'Keeffe, and E. P. Aserappa. Sixteen candidates entered for the respective divisions, and 9 passed.

### Medical Appointments.

THE GENERAL HOSPITAL, BIRMINGHAM.

